# Dissecting the mechanism of action of actinoporins. Role of the N-terminal amphipathic α-helix in membrane binding and pore activity of sticholysins I and II

**DOI:** 10.1371/journal.pone.0202981

**Published:** 2018-08-30

**Authors:** Gustavo P. B. Carretero, Eduardo F. Vicente, Eduardo M. Cilli, Carlos M. Alvarez, Håvard Jenssen, Shirley Schreier

**Affiliations:** 1 Department of Biochemistry, Institute of Chemistry, University of São Paulo, São Paulo, Brazil; 2 Department of Science and Environment, Roskilde University, Roskilde, Denmark; 3 Faculty of Science and Engineering, State University of São Paulo, Tupã, Brazil; 4 Institute of Chemistry, State University of São Paulo, Araraquara, Brazil; 5 Center for Protein Studies, University of Havana, Havana, Cuba; Rijksuniversiteit Groningen, NETHERLANDS

## Abstract

Actinoporins sticholysin I and sticholysin II (St I, St II) are proposed to lyse model and biomembranes via toroidal pore formation by their N-terminal domain. Based on the hypothesis that peptide fragments can reproduce the structure and function of this domain, the behavior of peptides containing St I residues 12–31 (StI_12-31_), St II residues 11–30 (StII_11-30_), and its TOAC-labeled analogue (N-TOAC-StII_11-30_) was examined. Molecular modeling showed a good match with experimental structures, indicating amphipathic α-helices in the same regions as in the toxins. CD spectra revealed that the peptides were essentially unstructured in aqueous solution, acquiring α-helical conformation upon interaction with micelles and large unilamellar vesicles (LUV) of variable lipid composition. Fluorescence quenching studies with NBD-containing lipids indicated that N-TOAC-StII_11-30_’s nitroxide moiety is located in the membranes polar head group region. Pyrene-labeled phospholipid inter-leaflet redistribution suggested that the peptides form toroidal pores, according to the mechanism of action proposed for the toxins. Binding occurred only to negatively charged LUV, indicating the importance of electrostatic interactions; in contrast the peptides bound to both negatively charged and zwitterionic micelles, pointing to a lesser influence of these interactions. In addition, differences between bilayers and micelles in head group packing and in curvature led to differences in peptide-membrane interaction. We propose that the peptides topography in micelles resembles that of the toxins in the toroidal pore. The peptides mimicked the toxins permeabilizing activity, St II peptides being more effective than StI_12-31_. To our knowledge, this is the first demonstration that differences in the toxins N-terminal amphipathic α-helix play a role in the difference between St I and St II activities.

## Introduction

Sticholysin I and sticholysin II (St I and St II), cytolysins produced by the sea anemone *Stichodactyla helianthus*, belong to the family of actinoporins [1, 2]. These toxins present 93% sequence homology and act by forming pores in membranes, their putative receptor being the phospholipid sphingomyelin. However, the various steps of membrane binding, oligomerization, and pore formation are not completely understood at the molecular level. St I and St II differ by thirteen amino acids, of which only three non-conserved residues are located in the N-terminus (Glu^2^/Ala^1^, Asp^9^/Ala^8^, Gly^23^/Glu^22^) [1, 2, 3]. As a result, St II displays higher hemolytic activity than St I (30,000 units of hemolysis/mg and 21,700 units of hemolysis/mg, respectively) [1, 2, 3].

High resolution solution studies—X-ray crystallography of St II [4] and NMR of St I [5]—indicated that the proteins are folded as a β-sandwich flanked by two α-helical segments, one in the N-terminal region and the other in the middle of the polypeptide chain. In both cases the first 13 residues are essentially unordered, except for residues 4–8 of St II, which are in β-sheet conformation. Moreover, this portion of St II is much more hydrophobic than its counterpart in St I. In both toxins this region is followed by a segment in amphipathic α-helical conformation, comprising eleven residues (15–25 in St I and 14–24 in St II). Circular dichroism (CD) [6, 7] and Fourier Transform Infrared spectroscopies (FTIR) [8] of St I and St II also showed a high β-sheet content and a low content of α-helical secondary structure. Other actinoporins, equinatoxin II (Eqt II) [9, 10] and fragaceatoxin (FragC) [11] present similar three-dimensional folds.

CD and FTIR studies also showed that binding to model lipid bilayers leads to an increase in St I, St II, and Eqt II α-helical content [6–8, 12]. The major conformational change seems to take place in the N-terminal region, with expansion of the existing α-helix towards the N-terminus [13], while the β-sandwich and the second helical segment would retain essentially the same structure as in solution and take part in membrane recognition (binding) and scaffolding on the bilayer surface [4].

After membrane binding, the N-terminal helix is proposed to be released from the protein core (β-sandwich) and to interact with the membrane in an orientation approximately parallel to the bilayer surface, at the interface. In further steps, two or more sticholysin monomers would promote lipid reorganization, giving rise to a toroidal pore. Mutation and truncation studies have led to the proposal that the N-terminal region of actinoporins is the proteins active part in pore formation [14–18], the amphipathic α-helical segment being the region that forms the pore channel, while the first residues would anchor the protein in the membrane acyl chain region [1, 4, 8, 16, 19]. The hydrophilic pore lumen (1 nm) [20] would then be formed by the helix hydrophilic face intercalated with the lipids polar head-groups in positive curvature topography [4, 7, 13, 18, 19]. Although it is often proposed that the pore consists of a tetramer, pores as small as a dimeric species have been suggested. More recently, based on single channel current measurements, it has been proposed that there is no fixed stoichiometry behind the formation of these oligomeric pores, with a distribution of the number of monomers giving rise to the pores [21, 22]. In contrast to what is proposed for St I, St II, and Eqt II, recent X-ray crystallography and cryo-electron microscopy studies showed the formation of a nonameric α-helical barrel-stave-like pore for FragC [11], with small lipid participation [23].

Based on the hypothesis that protein fragments are capable of mimicking their conformation (and function) in the whole protein, synthetic peptides corresponding to the N-terminal sequences of both sticholysin isoforms have been used to better understand the role of this region in pore formation [5, 24–28] and Eqt II [29, 30]. Similarly to the toxins, peptide segments comprising residues 1–31 and 1–30 of St I and St II, respectively, showed hemolytic activity, albeit at much higher concentrations. The peptides were capable to acquire amphipathic α-helical conformation in membrane-mimetic systems (2,2,2-trifluoroethanol (TFE) and model membranes), and the helical content was higher in these media when compared to that found for the proteins high resolution structure in solution. Moreover, the pore formed by the StII_1-30_ peptide was similar in size to that formed by St II [24].

To gain further insight into the molecular mechanism of pore formation, we report the synthesis of peptides comprising the N-terminal amphipathic α-helical segments of St I and St II. The peptides correspond to residues 12–31 of St I (StI_12-31_) and 11–30 of St II (StII_11-30_). A third peptide, bearing the amino acid spin label 2,2,6,6-tetramethyl-N-oxyl-4-amino-4-carboxylic acid, TOAC [31] at the N-terminus of StII_11-30_ (N-TOAC-StII_11-30_) was also synthesized (Table 1). A comprehensive spectroscopic study was performed aiming at obtaining information concerning model membrane-peptide interaction, as well as the peptides ability to affect the permeability of model and biological membranes. Binding and affinity of the peptides for model membranes–vesicles and micelles–of variable lipid composition were examined, as well as their conformational properties in solution and upon binding. The peptides membrane location was also investigated. Pore forming activity was assessed by model membrane leakage and hemolysis assays. The structure-dynamics-function results are discussed in the light of the role of the toxins N-terminal amphipathic helix in pore formation.

**Table 1:** Peptides sequence, formal charge at pH 7.0, and molecular weight.

Peptide	Sequence	Charge at pH 7	Molecular weight
StI_12-31_	^12^SLTFE VLDKV LGELG KVSRK^31^	+2	2,217.6 Da
StII_11-30_	^11^SLTFQ VLDKV LEELG KVSRK^30^	+2	2,288.7 Da
N-TOAC-StII_11-30_	TOAC ^11^SLTFQ VLDKV LEELG KVSRK^30^	+1	2,503.7 Da

## Materials and methods

### Reagents

The phospholipids 1-palmitoyl-2-oleoyl-sn-glycero-3-phosphocholine (POPC), 1,2-dipalmitoyl-sn-glycero-3-phosphocholine (DPPC), 1-palmitoyl-2-oleoyl-sn-glycero-3-phosphate (sodium salt, POPA), 1,2-dimyristoyl-sn-glycero-3-phosphate (sodium salt, DMPA), bovine brain sphingomyelin (SM), 1-palmitoyl-2-hydroxy-sn-glycero-3- phosphocholine (lyso-PC, LPC), 1-palmitoyl-2-hydroxy-sn-glycero-3-phosphate (sodium salt, lyso-PA, LPA), lysosphingomyelin (LSM), and the fluorescently labeled lipids 1,2-dipalmitoyl-sn-glycero-3-phosphoethanolamine-N-(7-nitro-2-1,3-benzoxadiazol-4-yl) (ammonium salt, DPPE-NBD), 1-palmitoyl-2-{6-[(7-nitro-2-1,3-benzoxadiazol-4-yl)amino]hexanoyl}-sn-glycero-3-phosphocholine (PC-6-NBD), 1-palmitoyl-2-{12-[(7-nitro-2-1,3-benzoxadiazol-4-yl)amino] dodecanoyl}-sn-glycero-3-phosphocholine (PC-12-NBD) and 1,2-dioleoyl-sn-glycero-3-phosphoethanolamine-N-(1-pyrenesulfonyl) (ammonium salt, DOPE-Pyr) were purchased from Avanti Polar Lipids (Alabaster, AL, USA), and used without further purification.

### Peptide synthesis

C-terminal amidated peptides (Table 1) were synthesized manually according to the standard Nα-Fmoc protecting group strategy [32], as previously described [24]. The peptides homogeneity and identity was checked by analytical HPLC-MS in an LTQ XL Linear Ion Trap Mass Spectrometer (ThermoFisher Scientific, Waltham, MA, USA); amino acid analysis was performed in a Shimadzu model LC-10A/C-47A amino acid analyzer (Shimadzu, Tokyo, Japan). The peptides purity was: StI_12-31_, 98%, StII1_1-30_, 95%, N-TOAC-StII_11-30_, 96%. The numbering of amino acids residues in the peptides is the same as in the toxin sequence.

### Model membranes

Stock solutions were prepared by weighing and dissolving the lipids in chloroform:ethanol mixtures of variable proportions. Lipid films were obtained by evaporating the solvent under a stream of nitrogen and subjecting the film to vacuum for two hours. Large unilamellar vesicles (LUV) were prepared by resuspending the lipids in 2.5 mM phosphate-borate-citrate (PBC) buffer, pH 7.0. The suspension was freeze-thawed six times and LUV were obtained by extrusion through two stacked polycarbonate filters (100 nm pore size, Nuclepore, Maidstone, UK). Micelles were obtained by dissolving the lysophospholipids in 5.0 mM PBC buffer.

### TOAC location in the bilayer—NBD fluorescence quenching

N-TOAC-StII_11-30_, in the concentration range 0–60 μM, was added to 50 μM DPPC:DMPA 90:10 (mole%) LUV containing NBD-labeled lipids (Lip-NBD, 1% of total lipids, in moles).Samples were placed in quartz cuvettes and NBD fluorescence quenching by the nitroxide moiety was monitored by measuring the emission between 475 nm and 675 nm (λex 469 nm) in a Hitachi F-4500 spectrofluorimeter (Hitachi, Japan). The maximum emission intensity was recorded in each condition, normalized by the NBD maximum emission in the absence of peptide (F_0_/F_P_) and analyzed as a function of peptide concentration and NBD position in the phospholipid molecule.

### DOPE-Pyr redistribution between outer and inner bilayer leaflets

Asymmetric incorporation of DOPE-Pyr into LUV was performed by drying an aliquot of DOPE-Pyr stock solution corresponding to 5% (in moles) of total lipid, resuspending the film in 10 μL ethanol, and adding 1mL LUV containing100 μM POPC:POPA 85:10 (mole %). The preparation was then placed in a water bath at 37°C for 30 min. Incorporation of DOPE-Pyr was monitored by measuring pyrene fluorescence emission at 450 nm until it was stable (approx. 40 min), using 344 nm excitation wavelength. Emission was registered in a FlexStation 3 Multi-mode Microplate Reader (Molecular Devices, Sunnyvale, CA). Measurements were made before and after addition of increasing peptide concentrations, and after addition of 0.1% Triton-X100.

The redistribution of the Pyr-labeled lipid between the bilayer outer and inner monolayers after 25 minutes (% Redistribution) was monitored by the decrease of excimer emission (F_25_) and was normalized by the initial emission at 450 nm (in the absence of peptide, F_0_) and the emission after Triton-X100 addition (F_Max_):

$$\%\mathrm{Redistribution}=100\;x\frac{\left(F_{0}‑F_{25}\right)}{\left(F_{0}-F_{\mathrm{Max}}\right)}$$ (1)

### Peptide-induced membrane permeabilization–Carboxyfluorescein leakage

LUV were prepared in a solution of 50 mM carboxyfluorescein (CF, Eastman Kodak, Rochester, NY) in 20 mM Tris-HCl, pH 8.0. The untrapped fluorophore was separated from the LUV by gel-filtration on a pre-packed Sephadex G-25 mini-column (GE Healthcare, Buckinghamshire, UK) equilibrated with 20 mM Tris-HCl and 300 mM NaCl, pH 8,.0. Lipids were quantified according to Rouser et al. [33].

The increase in CF fluorescence emission at 520 nm due to LUV permeabilization (20 μM total lipid concentration) was recorded as a function of time in a FlexStation 3 Multi-mode Microplate Reader, exciting the sample at 490 nm. The total CF release after 50 min (F_P_) was normalized by the initial emission (in the absence of peptide, F_0_), and the maximum emission by adding 0.1% Triton-X100 (F_Max_):

$$\%\mathrm{CF}\;\mathrm{Release}=100\;x\frac{\left(F_{P}‑F_{0}\right)}{\left(F_{\mathrm{Max}}-F_{0}\right)}$$ (2)

The total release (%) after 50 min was studied as a function of peptide concentration and Eq 3 was adjusted to the experimental data to obtain C_50_, the peptide concentration that causes 50% release of encapsulated CF.

$$\%\mathrm{CF}\;\mathrm{Release}=100‑\frac{100}{1+([\mathrm{Pep}]/C_{50}{)}^{n}}$$ (3)

This study yielded the [Peptide]/[Lipid] ratio that causes 50% release (P/L)_50_, and the Hill cooperativity coefficient (n).

### Peptide hemolytic activity

Five milliliters of fresh red blood cells (RBC) from healthy volunteers were mixed with 40 mL sterile saline solution (0.9% NaCl in water), centrifuged at 1500 rpm (500 g) for 10 min, and the supernatant was removed. The washing process was repeated three times until the supernatant was clear after centrifugation. Two milliliters of the pellet were mixed with 8 mL saline solution to obtain a 20% (in volume) RBC suspension. The peptides were tested in triplicate at concentrations ranging from 2 μM to 128 μM in a two-fold serial dilution. To start the assay, 50 μL of peptide from the serial dilution in saline solution were mixed with 50 μL of 20% RBC, resulting in a 10% RBC final concentration. The experiment was carried out in COSTAR 96-well polypropylene microplates (No.3879, Corning, NY, USA). Positive and negative controls were a 0.1% Triton X-100 solution in a 10% RBC suspension, to cause 100% cell lysis, and a 10% RBC suspension in the absence of peptides, respectively. The plates were incubated at 37°C for 24 h and centrifuged at 1200 rpm (400 g) for 10 min. Twenty microliters of the supernatant were diluted in 100 μL saline and the absorption was measured at 414 nm and 546 nm in a Bio-Tek Synergy HT Microplate Reader (Winooski, VT, USA); measurements were done in polystyrene flat bottom 96-well microplates (Greiner Bio-one, Kremsmünster, Austria).

Hemolysis (%) was calculated using absorbance values of the samples in the presence of peptide (A_PEP_), the positive (A_P_) and negative (A_N_) controls making use of Eq 4:

$$\mathrm{Hemolysis}=100\;x\frac{\left(A_{\mathrm{PEP}}-A_{N}\right)}{\left(A_{P}-A_{N}\right)}$$ (4)

The approval for the work making use of human red blood cells was granted by “Arbeids Tilsynet” in respect to “genteknologiske Forskningsprojekter” dated 11/11/2014 and it is valid for 5 years. Additionally the blood work has been carried out in accordance with internal safety and ethics regulations approved by Stine Korreman at Roskilde University.

### CD measurements

CD spectra were obtained in a Jasco J-720 Spectropolarimeter (Jasco, Japan). Samples were placed in 1.00 mm optical length quartz cells. The final spectra were the average of 6 scans, following subtraction of the spectrum obtained under the same conditions of a sample without peptide. Spectra were scanned from 190 nm to 260 nm, at 50 nm/min, using a 2 nm slit. The initial peptide concentration was 12 μM. Lysophospholipid and phospholipid concentrations varied from 0 to 10 mM and from 0 to 0.8 mM, respectively.

### Analysis of CD spectra

Analyses of peptide binding experiments were carried out normalizing the intensity of the molar residual ellipticity [θ] at 222 nm at different lipid concentrations using Eq 5, and fitting Eq 6 to the experimental data:

$$[\theta {]}_{\mathrm{Norm}}=\frac{\left([\theta {]}_{222}-[\theta {]}_{2220}\right)}{\left([\theta {]}_{222\mathrm{Max}}-[\theta {]}_{2220}\right)}$$ (5)

$$[\theta {]}_{\mathrm{Norm}}=1+([\theta {]}_{\mathrm{NormMax}}-1)x\frac{\left[\mathrm{Lipid}\right]}{\left(1/K_{B})+[\mathrm{Lipid}\right]}$$ (6)

where [θ]_222_, [θ]_2220_, and[θ]_222Max_ correspond to the peptide molar residual ellipticities at different lipid concentrations, in solution, and in the presence of 0.8 mM LUV or 10 mM micelles, respectively. [Lipid] corresponds to the lipid concentration (mol/L), and K_B_, corresponds to the apparent binding constant (M^-1^). Spectral deconvolutions were performed using the PEPFIT program [34] and a set of standard curves taken from Greenfield and Fasman [35].

### In silico peptide structural analysis

The peptides were modeled using the PEP-FOLD software available online (http://mobyle.rpbs.univ-paris-diderot.fr/cgi-bin/portal.py#forms::PEP-FOLD) [36, 37, 38]. By uploading the amino acid sequence, the platform initially performs a local conformational prediction based on a Structural Alphabet, describing the probability of each fragment of four consecutive residues to adopt a given conformation, and associating a Structural Alphabet letter to describe the conformation of the backbone angles of each fragment. The Structural Alphabet profile is then processed to select a limited number of local conformations. Finally, the software combines the calculated fragments to produce a full model structure followed by a coarse-grained simulation [36, 37, 38]. The models are post-treated by replacing coarse-grained side chain beads by all atom side chains, followed by a fast energy minimization performed with the GROMACS program [39]. A cluster of five structures ranked with the lowest energy is then retrieved in a .pdb file format.

### Structure comparison

The five structures obtained from PEP-FOLD for each peptide were superimposed with the model of St I (PDB code: 2KS4) [5] or St II (PDB code: 1GWY) [6] using UCSF Chimera (http://www.cgl.ucsf.edu/chimera) [40]. The peptide models exhibiting the lowest backbone RMSD values when compared to the toxins were selected for further analysis. Molecular graphics and visualization were performed using PyMol Educational Version 1.3 (https://www.pymol.org/) and Visual Molecular Dynamics 1.9.2 (http://www.ks.uiuc.edu/Research/vmd/vmd-1.9.2/).

## Results

### In silico structural analysis

**Fig 1:**
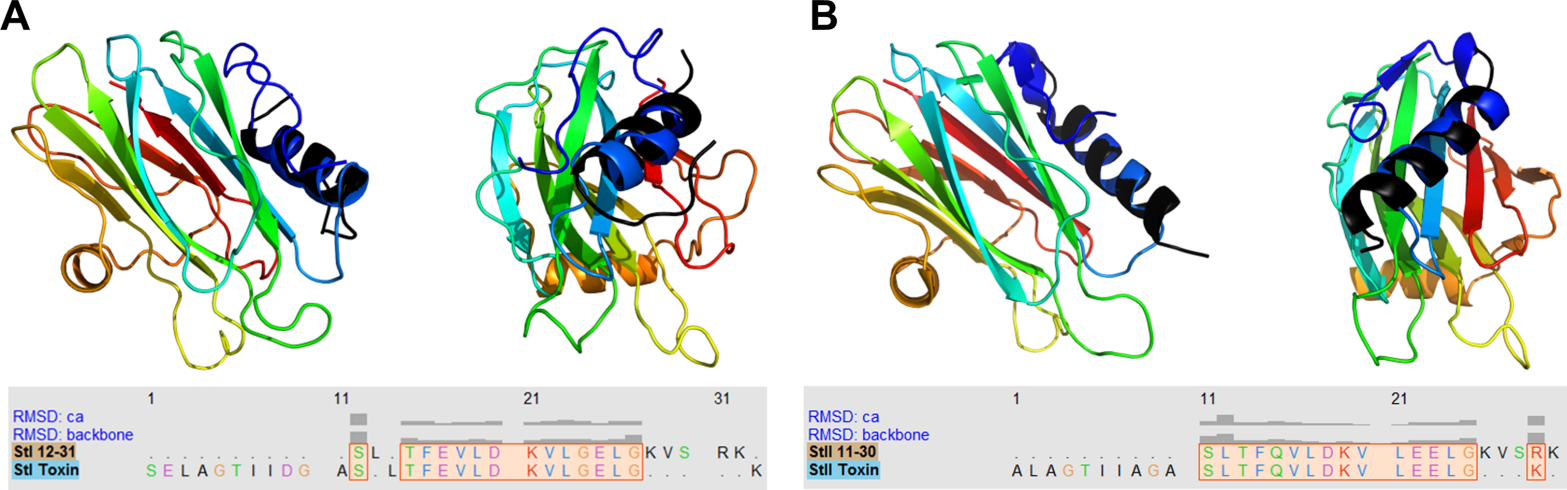
Peptides modeled structures match toxins high resolution structures. Superposition of the rainbow-colored structure of St I (A) [5] and St II (B) [4], with the black-colored structure of StI_12-31_ and StII_11-30_ obtained by the PEP-FOLD program, respectively. Each toxin is shown at two orientations. Peptide and toxin sequence alignment and bars of RMSD values (between 0 and 1 Å) of the superimposed structures. RMSD > 1 not shown.

The structures of both modeled peptides, StI_12-31_ and StII_11-30_, match the experimentally found structures of the same regions—obtained by NMR for St I [5] and from crystallographic data for St II [4]. In StI_12-31_ and in its corresponding toxin, the region between residues F^15^-L^25^ adopts a α-helical conformation, while the regions flanking the helical segment present no defined secondary structure (Fig 1A). Likewise, the modeled StII_11-30_ peptide presents α-helical conformation in the F^14^-L^24^ region (Fig 1B), as St II. The superposition of the modeled and experimental structures performed using the UCSF Chimera software [40] shows a good fit, especially in the regions comprising residues 12–26 (StI_12-31_) and 11–25 (StII_11-30_), where the Cα and backbone calculated RMSD are less than 1 Å (Fig 1A and 1B). Major differences between the structures of the model peptides and the toxins are located in the regions comprising residues 26–31 (St I) and 25–30 (St II). In the toxins, these regions present a flexible stretch preceding the first β-sheet core segment, while the modeled structures present one more helical turn (Fig 1). The influence of the rest of the sequence forming the β-sheet core upon the above segments in the toxins is probably responsible for the observed differences.

**Fig 2:**
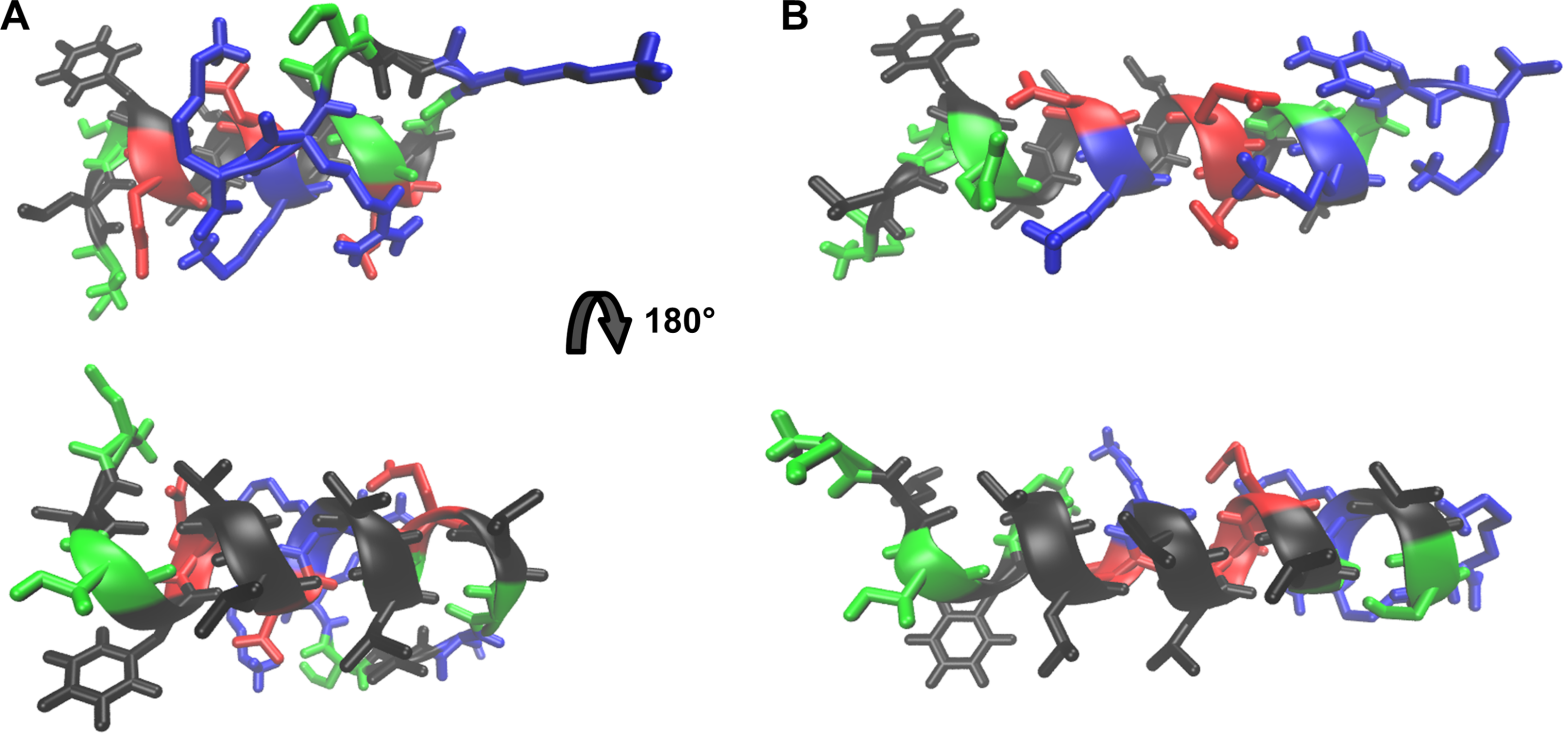
Atomic representation of the models of peptides StI_12-31_ and StII_11-30_ obtained by the PEP-FOLD program. StI_12-31_ (A) and StII_11-30_ (B) structures. Colors of residues types: Non-polar (black), polar uncharged (green), negatively charged (red) and positively charged (blue).

The α-helical portions of both modeled peptides display hydrophobic (black, Fig 2) and hydrophilic (green, blue and red, Fig 2) faces. It is postulated that, alongside with the stretches corresponding to the toxins first residues, this region promotes lipid bilayer reorganization to form a toroidal pore where the helices hydrophilic face would line-up the pore´s cation selective core [20], while the hydrophobic face would interact with the lipid acyl chain region.

Salt bridges between positively and negatively charged side chains in consecutive α-helix turns can be observed in both StI_12-31_ and StII_11-30_ high resolution models. In Fig 2, it is possible to observe ion pair formation between residues E^16^ and K^20^ in StI_12-31_, while in StII_11-30_, ion pair formation is observed between residues E^22^ and K^26^. Other calculated structures of both St I and St II N-termini reveal additional possibilities of ion pair formation: in StI_12-31_ (K^20^ and E^24^ and E^24^ and K^27^), and in StII_11-30_ (K^19^ and E^22^, K^19^ and E^23^, and E^23^ and K^26^) (S1 Fig).

The hydrophobic faces in the toxins helical segments contain uncharged polar residues at both ends (T^14^ and S^29^, in StI_12-31_ and T^13^ and S^28^, in StII_11-30_). It is conceivable that these residues would reside in the membrane hydrophobic/hydrophilic interface, modulating the helix insertion in the membrane and playing a role in defining the angles formed between the helix axis and the preceding residues, and between this axis and the β-sheet connecting the N-terminus to the body of the protein. In the pore region, several peptide molecules would promote membrane positive curvature by recruiting lipids that favor this molecular arrangement.

### CD conformational studies

**Fig 3:**
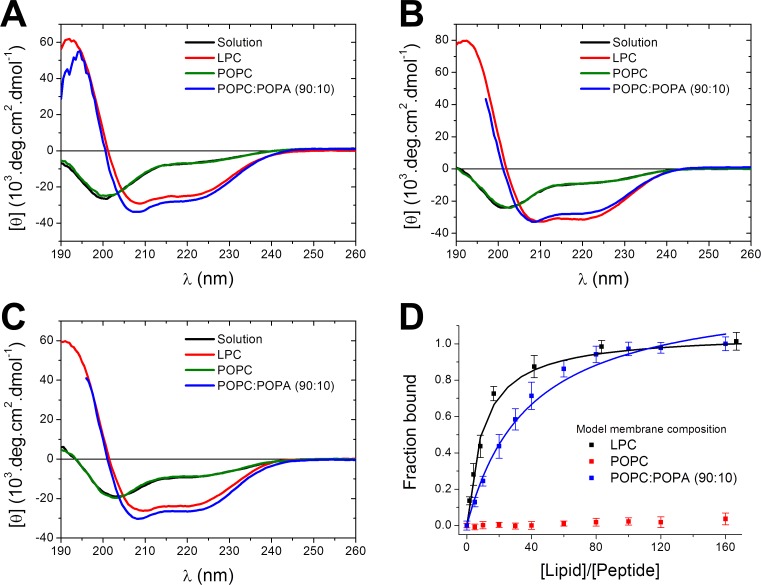
CD spectra of the peptides and binding isotherms. CD spectra of 12 μM StI_12-31_ (A), StII_11-30_ (B) and N-TOAC-StII_11-30_ (C) in solution and in the presence of 10 mM LPC micelles or 0.4 mM POPC or POPC:POPA (90:10) LUV, pH 7.0. (D) Peptide-membrane binding isotherms of StII_11-30_ obtained from normalized [θ] values as a function of [Lipid]/[Peptide] ratio.

In aqueous solution, all three peptides present CD spectra with a negative peak at 203 nm and a negative low intensity peak around 222 nm, indicating that the peptides are predominantly flexible, with low α-helical secondary structure content (Fig 3). Spectral deconvolution showed that StI_12-31_ presents 18% helical content (four residues), while StII_11-30_ and N-TOAC-StII_11-30_ are in approximately 25% α-helical conformation (five residues, Table 2).

**Table 2:** Peptides secondary structure content calculated from CD spectra using the PEPFIT program.

	Medium	% α-helix	% β-sheet	% Random	R^2^
StI_12-31_	Aqueous solution	18	7	75	0.921
	LPC Micelle	74	0	26	0.986
	POPC:POPA (90:10) LUV	80	0	20	0.942
	Modeled peptide	60	0	40	-—- -
StII_11-30_	Aqueous solution	23	0	77	0.960
	LPC Micelle	85	0	15	0.996
	POPC:POPA (90:10) LUV	77	0	23	0.988
	Modeled peptide	80	0	20	-—- -
N-TOAC-StII_11-30_	Aqueous solution	24	4	72	0.961
	LPC Micelle	72	0	28	0.992
	POPC:POPA (90:10) LUV	71	0	29	0.980

Conformational changes in the presence of lipids are interpreted as indicative of peptide-membrane interaction. In the presence of model membranes—micelles and bilayers of variable lipid composition–the spectra display an intense positive peak around 195 nm and two negative peaks at ca. 208 nm and 222 nm, indicative of α-helical conformation (Fig 3). Spectra deconvolution and calculation of secondary structure content showed that the bound forms of the three peptides possess between 75 and 85% of α-helical content, corresponding to 15–17 residues (Table 2). Varying the micelle lipid composition–whether the systems contained only zwitterionic lipids or additional negatively charged lysophosphatidic acid—did not alter the spectra, or the calculated secondary structure content to a significative extent (S2 Fig). In contrast, in the presence of LUV, the peptides only acquired α-helical conformation when the membranes contained negatively charged phosphatidic acid (Fig 3, Table 2).

The less water-available environment of micelles or bilayers favors the formation of peptide intramolecular hydrogen bonds [41], stabilizing the α-helical conformation. In the case of bilayers carrying zwitterionic surfaces, like POPC and POPC:SM, membrane addition did not trigger structural changes, indicating that the peptides remained in solution. StI_12-31_, StII_11-30_, and N-TOAC-StII_11-30_ interacted to a large extent with LUV containing negatively charged POPA (Fig 3, Table 3) demonstrating that binding to bilayers depends crucially on electrostatic interactions. In contrast, the peptides bound to a large extent to both zwitterionic and negatively charged micelles (Table 3). The bilayer-bound conformation of all three peptides was similar regardless the lipid system, indicating the high propensity of these sequences to acquire a α-helical conformation.

**Table 3:** Peptide binding to model membranes.

Membrane			
	StI_12-31_	StII_11-30_	N-TOAC-StII_11-30_
**LPC**	4	9	13
**LPC:LSM 90:10**	5	8	12
**LPC:LPA 90:10**	10	13	18
**LPC:LPA:LSM 80:10:10**	9	10	12
**POPC**	No binding	No binding	No binding
**POPC:SM 90:10**	No binding	No binding	No binding
**POPC:POPA 90:10**	1.2	1.7	1.5
**POPC:POPA:SM 80:10:10**	1.2	1.8	1.6

Values of the apparent binding constant, K_b_ (10^3^ x M^-1^), for the interaction of StI_12-31_, StII_11-30_, and N-TOAC-StII_11-30_ with micelles and bilayers calculated from CD experiments, pH 7.0.

The analysis of [θ]_222_ as a function of lipid concentration yielded binding isotherms (Fig 3D), allowing quantitative comparison of the peptides affinity for the different lipid systems. While StII_11-30_ and its TOAC-containing analogue showed essentially the same behavior in all studied conditions, StI_12-31_ yielded lower apparent binding constants (Table 3), suggesting that the sequence differences, namely, mutations E^16^→Q^15^ and G^23^→E^22^ when comparing StI_12-31_ and StII_11-30_, are capable of influencing peptide binding.

### NBD fluorescence quenching by TOAC

**Fig 4:**
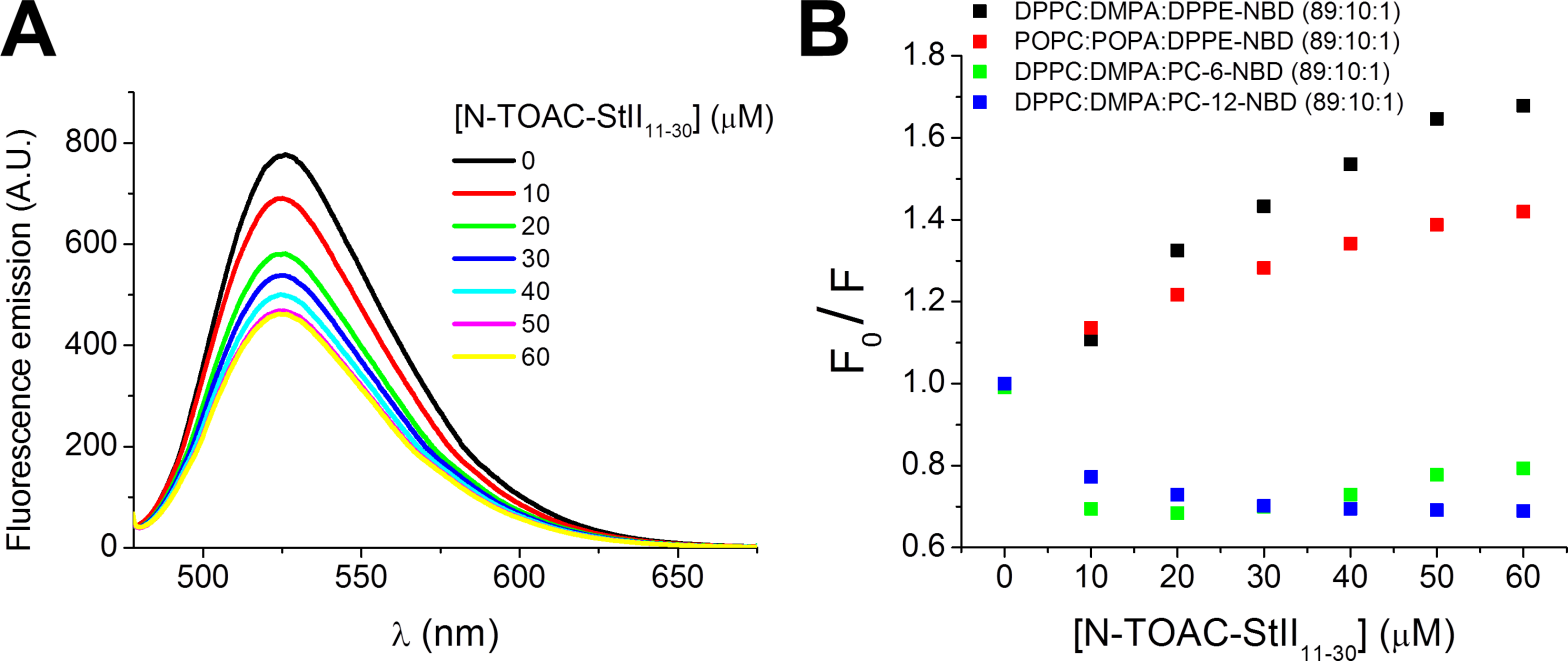
NDB fluorescence emission quenching by TOAC residue of N-TOAC-StII_11-30_. (A) Fluorescence emission spectra of 0.5 μM DPPE-NBD incorporated in 50 μM DPPC:DMPA (90:10) LUV in the presence of increasing concentrations of N-TOAC-StII_11-30_. (B) Normalized maximum fluorescence emission as a function of peptide concentration.

Fluorescence quenching properties of TOAC’s paramagnetic nitroxide group can be explored to study its position in the bilayer [31]. Energy transfer from a fluorophore excited state to a nitroxide occurs only upon contact between these groups [42]. Due to the close proximity required for quenching, the effect can be related to the relative position of both groups in the bilayer. In this study, phospholipids labeled in the head group (DPPE-NBD) and at carbons 6 (PC-6NBD) and 12 (PC-12-NBD) of the acyl chain (Experimental procedures) were used. For studies with the acyl chain-labeled probes, gel phase DPPC:DMPA:Lip-NBD (89:10:1) LUV were used to avoid flipping of the polar fluorophore moiety to the membrane-water interface [43]. The peptides conformation and binding to gel lipid phase membranes were previously checked by CD, yielding (Supporting Information 3) results similar to those found for liquid crystalline POPC:POPA (90:10) bilayers. In the case of DPPE-NBD fluorophore, both types gel and crystalline lipid systems were used, yielding similar results regarding the position of TOAC residue in the bilayer (Fig 4).

Fig 4 presents the fluorescence quenching data obtained for both gel and liquid-crystalline lipid systems. Quenching was much more effective when the NBD moiety was located in the lipid head group (Fig 4B), suggesting that the paramagnetic amino acid residue resides in the bilayer head group region. In studies with PC-6-NBD and PC-12-NBD, addition of the first peptide aliquots triggered an increase of the emission intensity (Fig 4B) and a shift of the maximum emission to lower wavelengths, possibly due to a peptide-promoted change in bilayer organization. Further addition led to slight and no fluorescence quenching for PC-6-NBD and PC-12-NBD, respectively. The small quenching effect upon PC-6-NBD fluorescence at higher peptide concentrations suggests that the peptide’s N-terminus might visit the C6 region occasionally, due to peptide-induced changes in bilayer organization.

### Lipid redistribution between outer and inner bilayer leaflets

DOPE-Pyr asymmetrically-labeled vesicles were studied to investigate the nature of the pore formed by the peptides. The formation of a toroidal pore with the lipids arranged in positive curvature and the polar head groups participating in the pore lumen is proposed for Sts and other actinoporins [1, 4, 8, 16, 17, 19, 20, 44, 45]. The lipids in positive curvature create a continuous region between the bilayer inner and outer leaflets, allowing for lipid redistribution by lateral diffusion. DOPE-Pyr at high concentration in one of the bilayer leaflets presents a high intensity of the excimer fluorescence spectrum, while, when this same population is redistributed between the two leaflets, the intensity of this characteristic peak decreases at the expense of the increased intensity of the monomer spectrum.

**Fig 5:**
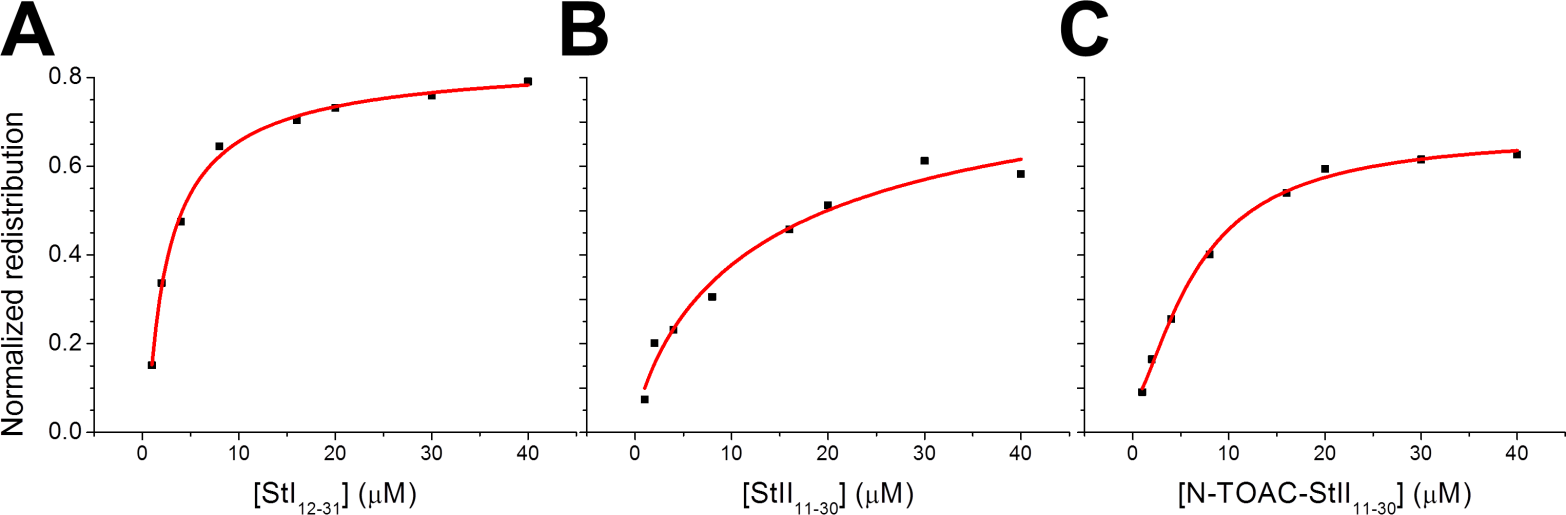
Lipid redistribution triggered by the peptides. Normalized redistribution of 5 μM DOPE-Pyr between bilayer leaflets of 100 μM POPC:POPA:DOPE-Pyr (85:10:5) in the presence of increasing concentrations of StI_12-31_ (A), StII_11-30_ (B) and N-TOAC-StII_11-30_ (C).

The addition of StI_12-31_, StII_11-30_, and N-TOAC-StII_11-30_ to LUV asymmetrically labeled with DOPE-Pyr (POPC:POPA:DOPE-Pyr (85:10:5)) triggered the redistribution of DOPE-Pyr, as seen by the decrease in excimer emission (Fig 5). The redistribution of the pyrene-labeled lipid occurred considerably faster than in the case of spontaneous flip-flop, suggesting that the peptides promote the formation of toroidal pores [46]. The extent of redistribution was concentration-dependent and essentially the same for all three peptides (Fig 5).

**Fig 6:**
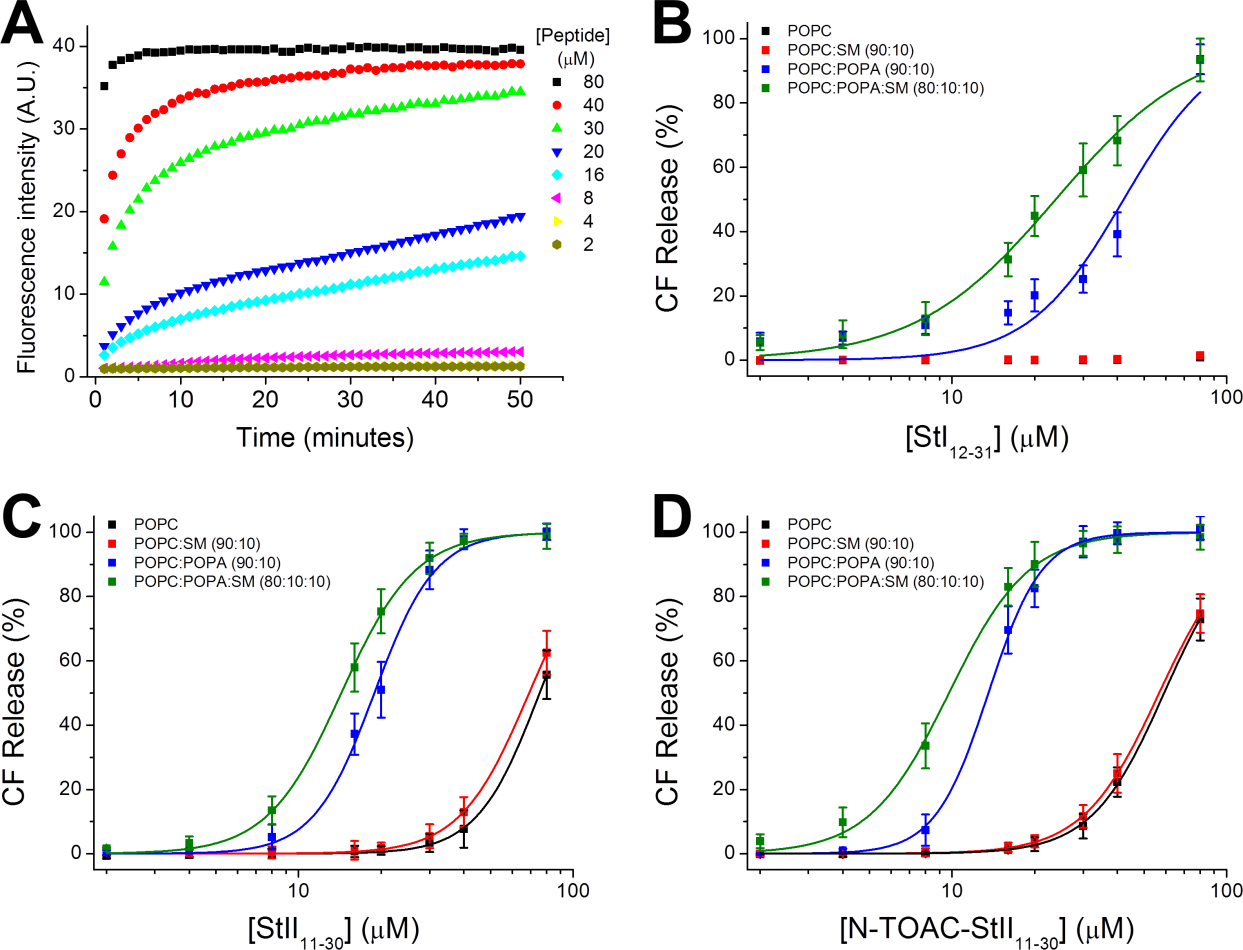
CF leakage from LUV triggered by peptide-membrane interaction. (A) Kinetics of CF release from 20 μM POPC:POPA (90:10) LUV triggered by increasing StII_11-30_ concentration. Total CF release after 50 minutes from LUV of variable lipid composition as function of peptide concentration. StI_12-31_ (B), StII_11-30_ (C) and, N-TOAC-StII_11-30_ (D). The lines represent fittings of Hill’s equation to the experimental data.

The occurrence of lipid mixing between the bilayer inner and outer monolayers does not necessarily imply toroidal pore formation. Total bilayer disruption, as in the carpet mechanism of action of bio-active peptides [47] would also result in lipid mixing between the two leaflets. Indeed, this was found in a study with the multi-functional peptide BP100 and two of its hydrophobic analogues [48]. Lipid mixing was observed; however, the kinetics of this process was different from that of leakage experiments. In conjunction with data for giant unilamellar vesicles (GUV), it was possible to associate lipid mixing to bilayer disruption, pointing to a carpet mechanism of action. Nevertheless, in the present case, lipid redistribution (Fig 5) occurred in a timescale similar to that of leakage of vesicle inner contents (Fig 6A, see below), which displayed a gradual pattern, compatible with a pore mechanism [49].

### Lipid vesicle permeabilization

The peptides ability to promote LUV permeabilization was assessed by the increase in CF fluorescence emission due to the loss of their internal aqueous content. Previous dye leakage assays showed that the full-length toxins, St I and St II, and their corresponding StI_1-31_ and StII_1-30_ fragments induced vesicle leakage in a lipid composition and peptide concentration-dependent manner [26–28]. In addition, the extent of leakage was greater for St II (and its corresponding peptide) than for St I (and its corresponding peptide). Similar results were found for StI_1-31_ and StII_1-30_ making use of the same lipid composition employed to study their shorter counterparts, StI_12-31_ and StII_11-30_ (Table 4). Activity was analyzed in terms of (P/L)_50_ values, i.e., the mole ratio of peptide to lipid required to obtain 50% of the maximum effect. The data indicate that the peptide concentrations required for activity against model lipid membranes are in the micromolar range, whereas the toxins were active in the nanomolar range [25, 28, 50]. However, if we take into account effective (P/L)_50_ values, estimated from the binding isotherms in Fig 3 (third column of Table 4), it is seen that peptide and toxin concentrations differ only by approximately one order of magnitude in the case of the St II peptides and are of the same order of magnitude for St I and its 12–31 peptide.

**Table 4:** CF leakage from LUV triggered by peptide-membrane interaction.

	(P/L)_50_	n	Effective (P/L)_50_ (% bound peptide)	Membrane composition	Reference
St I Toxin	0.0388		0.039 (100%)	PC:SM (50:50)	28
StI_1-31_	1.8	2.1		POPC:POPA (90:10)	This work
StI_12-31_	No activity			POPC	This work
	No activity			POPC:SM (90:10)	This work
	2.1	2.5	0.048 (2.3%)	POPC:POPA (90:10)	This work
	1.2	1.7	0.028 (2.3%)	POPC:POPA:SM (80:10:10)	This work
St II Toxin	0.0042		0.0042 (100%)	PC:SM (50:50)	28
StI_1-30_	0.15	2.7		POPC:POPA (90:10)	This work
StII_11-30_	3.7	3.8		POPC	This work
	3.4	3.4		POPC:SM (90:10)	This work
	0.91	3.9	0.030 (3.2%)	POPC:POPA (90:10)	This work
	0.71	3.2	0.024 (3.4%)	POPC:POPA:SM (80:10:10)	This work
N-TOAC-StII_11-30_	2.9	3.3		POPC	This work
	2.9	3.1		POPC:SM (90:10)	This work
	0.68	4.4	0.020 (3.0%)	POPC:POPA (90:10)	This work
	0.49	3.0	0.016 (3.2%)	POPC:POPA:SM (80:10:10)	This work

Toxin or peptide to lipid ratio, (P/L)_50_, required for leakage of 50% CF from LUV of variable lipid composition. n is the cooperativity coefficient.

Fig 6A shows CF release from POPC:POPA (90:10) LUV as a function of time for increasing StII_11-30_ concentration and Fig 6B, 6C and 6D show the total CF release after 50 min from LUV of variable lipid composition as a function of StI_12-31_, StII_11-30_, and N-TOAC-StII_11-30_ concentration, respectively. The lines represent fittings of Hill’s equation to the experimental data. All three peptides promoted CF leakage when exposed to LUV containing 10 mole % negatively charged POPA; addition of 10 mole % SM led to increased leakage (Fig 6B, 6C and 6D). Table 4 shows that the shorter peptides required higher (P/L) ratios than their longer counterparts for 50% effect on this same lipid system. In the case of zwitterionic POPC and POPC:SM (90:10) LUV, StI_12-31_ was unable to permeabilize these membranes in the concentration range studied (Fig 6B); in contrast, both StII_11-30_ and N-TOAC-StII_11-30_ acted upon POPC and POPC:SM (90:10) LUV, albeit at much higher concentrations than those acting against negatively charged LUV (Fig 6C and 6D).

(P/L)_50_ values and Hill coefficients resulting from analysis of the plots in Fig 6B, 6C and 6D are given in Table 4. StII_11-30_ and its paramagnetic analogue showed a greater permeabilizing effect than StI_12-31_; moreover, StII_11-30_ and its TOAC analogue displayed similar behavior. The sigmoidal shape of the leakage plots is strongly suggestive of a cooperative process. Interestingly, while Hill plot analyses in POPC:POPA LUV yielded n equal to 2.7 for StI_12-31_, this value was approximately 4 for StII_11-30_ and its N-TOAC analogue; furthermore, the n coefficient decreased in both cases when the membranes contained SM (Table 4).

### Hemolytic activity

**Fig 7:**
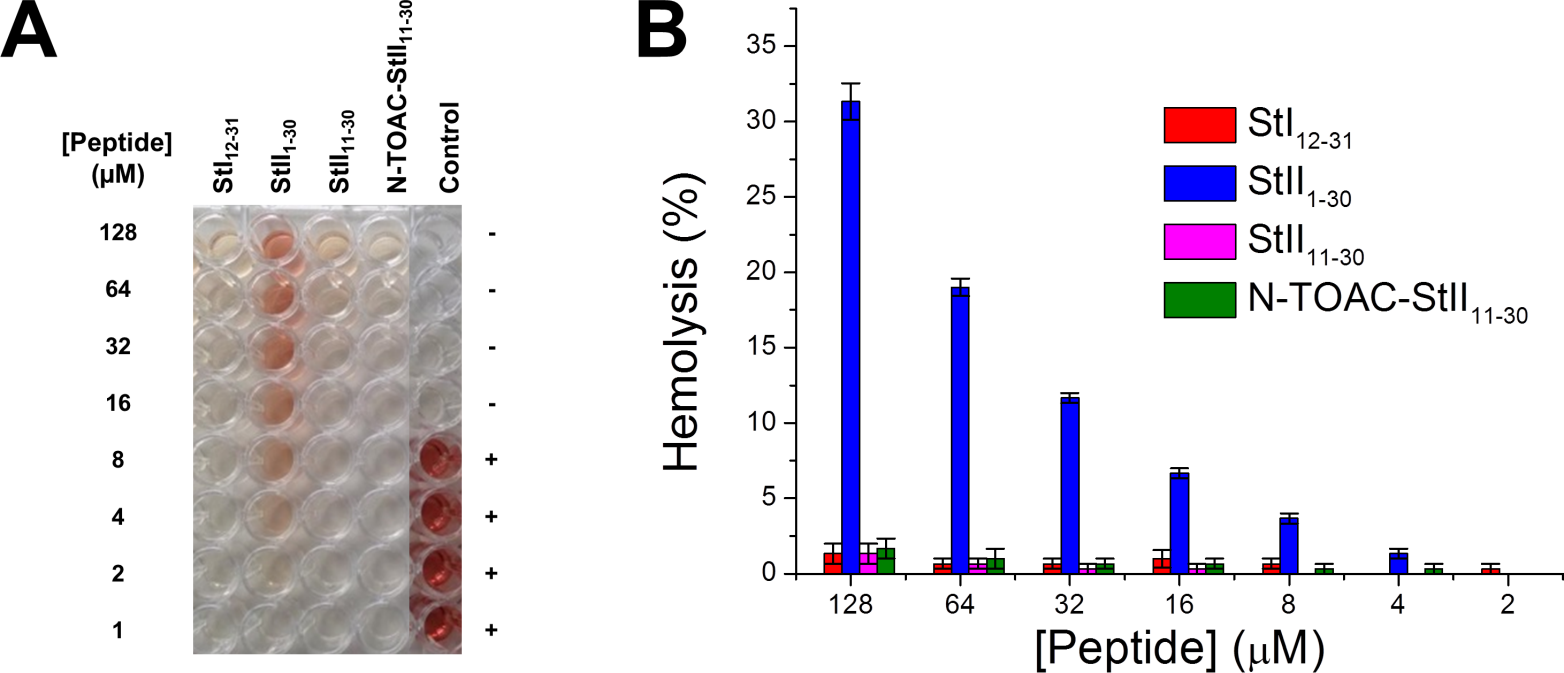
Hemolytic activity of the peptides. (A) Released hemoglobin from red blood cells by StI_12-31_, StII_1-30_, StII_11-30_ and N-TOAC-StII_11-30_. The last column displays the results for negative and positive controls. (B) Total percentage of hemoglobin released from red blood cells by the peptides.

The hemolytic activity of StI_12-31_, StII_11-30_, and N-TOAC-StII_11-30_ was compared to the already reported activities of the StI_1-31_ and StII_1-30_ fragments, as well as those of the full-length toxins. In previous studies it was found that, while the toxins were able to lyse RBC, at concentrations in the nanomolar range, only StII_1-30_ was able to lyse RBC, at micromolar concentrations [24, 25, 28]. In the present work it was found that, although being able to bind and promote LUV permeabilization (Fig 6, Table 4), the shorter peptides showed essentially no hemolytic activity up to 128 μM (Fig 7). The different behavior of StII1-30and StII_11-30_ highlights the importance of the 1–10 segment and its hydrophobic properties [25, 27, 28]. This is also stressed by the difference between the full length StI_1-31_ and StII_1-30_ peptides, since the former, whose 1–10 stretch is more charged, has a much lower hemolytic activity [24, 28].

## Discussion

### Modeling of peptide structure

Early studies of actinoporins aiming at elucidating their mechanism of action have proposed that these sea anemone toxins bind to membranes, promoting lysis via formation of toroidal pores, and implied their N-terminal domain in pore formation [44]. High resolution conformational studies of sticholysins, Eqt II, and FragC [4, 5, 10, 11, 23, 51] show structural similarities between their folds, including the N-terminal region. All of them present a more, or less, hydrophobic stretch (residues 1–11 in St I and 1–10 in St II) followed by a region with a high propensity to acquire an amphipathic α-helical conformation.

The structural models obtained for StI_12-31_ and StII_11-30_ (Fig 2) are in agreement with their expected conformation and match the experimental structures found for these fragments. In the models, the amphipathic peptides clearly display the respective polar and non-polar faces (Fig 2). This feature is known to be important for a large variety of membrane-active peptides [52, 53]. Polar, non-charged, residues located at both ends of the hydrophobic face–T^14^ and S^29^, in StI_12-31_ and T^13^ and S^28^ in StII_11-30_ –could be responsible for modulating the insertion of this segment in the lipid acyl chain region and influence its positioning and angle in the membrane and in the pore region. In addition, the initial 1–11 (1–10) residues and the whole β-sheet sandwich body of the toxins must also participate in the adequate positioning of the proteins in the membrane during the pore forming steps.

The energy minimization simulation performed by PEP-FOLD using GROMACS force-field [36] indicates formation of salt bridges between positive and negative side chains in successive turns of the helical segment. In Fig 2 salt bridges can be seen between E^16^ and K^20^ in StI_12-31_, and E^22^ and K^26^ in StII_11-30_. Salt bridges have been demonstrated to stabilize amphipathic membrane-active peptide structures [54, 55] and also play an important role in stabilizing helical conformations by increasing the energy necessary to disrupt each turn of the segment [56, 57].

Together with the other salt bridges observable in other calculated structures (see Results and S1 Fig), one can envision that these interactions can create a dynamic network of electrostatic interactions that contribute to the energetics of helix stabilization, especially in the less polar bilayer environment. It is worth noting the difference in number and nature of ion pairing in StI_12-31_and in StII_11-30_: while the St I stretch presents the ion pair forming side chains spread between residues 16 and 27, being able to establish a maximum of three ion pairs, in St II these side chains are located between residues 19 to 26, being able to form four ion pairs. The fact that less ion pairing is possible in StI_12-31_ may play a role in the lesser secondary structure content (Table 2), decreased membrane binding (Table 3), and reduced membrane permeabilizing activity (Table 4) found for this peptide. These properties are also probably related to the decreased lytic activity of the whole toxin St I when compared to St II [1–3, 28]. Moreover, charge distribution along the channel due to formation of intra- and inter-molecular side chain-side chain and side chain-lipid polar head group salt bridges in the final pore structure should contribute to the energetics of pore stabilization and could influence the pore’s cation selectivity [16, 19] and how these ions accumulate and flow through the pore’s lumen.

### Peptides conformation and dynamics in solution

Despite the high helical content observed in the 12–31 and 11–30 regions of St I and StII, respectively, with α-helices spanning their 15–25 and 14–24 segments, respectively [4, 5], both peptides showed only about five residues in this conformation in solution (Fig 3). This is due to lack of intramolecular hydrogen bonding between the peptide carbonyl and amino groups in view of the high content of water molecules competing for hydrogen bond formation with those groups. Accordingly, N-TOAC-StII_11-30_ presented low α-helical content (Fig 3).

### Peptide-micelle interaction

The peptides interacted with micelles of variable lipid composition, undergoing a considerable increase of α-helical structure (Fig 3, Table 2). The fact that the peptides bound to zwitterionic LPC and LPC:LSM 90:10 micelles (Fig 3, Table 3) indicates that hydrophobic interactions between the peptide helix nonpolar face and the hydrocarbon chains environment contribute to the energetics of peptide-micelle interaction. Furthermore, weak van der Waals interactions and hydration forces should also be considered. Last but not least, the intermolecular spacing between lipids due to micellar curvature most likely plays an important role in peptide binding to zwitterionic micelles. When negatively charged LPA was included, the binding constants increased for all three peptides (Table 3), indicating that electrostatic forces provided additional contribution to peptide binding. It is noteworthy that, while binding occurred to a considerable extent in the case of zwitterionic micelles, this interaction was very weak in the case of zwitterionic bilayers (Table 3).

### Peptide-bilayer (LUV) interaction

Peptides interaction with bilayers was verified by CD (Fig 3), by fluorescence quenching of NBD-labeled lipids by N-TOAC-StII_11-30_ (Fig 4), in the study of DOPE-Pyr transmembrane movement (Fig 5), and by activity assays (Fig 6). CD spectra showed that binding to LUV promoted conformational changes (Fig 3), namely, the peptides acquired α-helical structure, as observed in the presence of micelles. The fact that the bound conformation is similar in LUV of different lipid composition, as well as in different micelles, indicates the propensity of the peptides sequences to acquire a helical structure. Transfer to an environment with less water available to form hydrogen bonds with the peptide amide groups renders the peptide backbone more capable of establishing intramolecular hydrogen bonds, stabilizing the helical secondary structure [41].

High resolution studies of actinoporins in solution (or crystallized from solution) have pointed that the amphipathic α-helix spans about ten residues [4, 10, 11]. Furthermore, an approximately 5% increase in St II’s helical content (approx. 9 residues) was observed by CD [6, 7] and FTIR [8] upon binding to membranes. Table 2 shows that 15 to 17 residues are in α-helical conformation in the peptides in the presence of both micelles and LUV, suggesting that this region constitutes the major contribution to the increase in α-helical conformation when the full-length toxins bind to membranes.

### TOAC location in bilayers

NBD fluorescence quenching by N-TOAC-StII_11-30_ enabled the study of the paramagnetic moiety localization in the bilayer. The phenomenon was observed to a large extent for the head group-labeled lipid DPPE-NBD, less for PC-6-NBD and not at all for PC-12-NBD (Fig 4). Quenching of DPPE-NBD fluorescence in DPPC:DMPA LUV corroborates the results observed in liquid crystalline POPC:POPA LUV. In both systems the fluorophore is located at the expected position, and the data indicate that the paramagnetic probe in the peptide’s N-terminus lies in the bilayer polar head group region. The superficial location of the amphipathic α-helix was also demonstrated by NMR studies of the binding of St II´s fragment containing residues 16–35 to sodium dodecyl sulfate micelles [58]. In this study, hydrogen exchange data for StII_1-30_ pointed to the penetration of the 15–30 stretch into the hydrophobic part of the micelle. This result, however, is in contrast with fluorescence and fluorescence quenching data for tryptophan-containing analogues of this peptide in phospholipid bilayers, which suggest that the amphipathic α-helix is located at the membrane interface [27].

### Toroidal pore formation is suggested by peptide-induced lipid redistribution between bilayer leaflets

Actinoporins are proposed to form toroidal pores in membranes, inducing lipid positive curvature. According to this model, lipid redistribution should occur between the outer and inner bilayer leaflets. This phenomenon was observed in the study with the fluorescent probe DOPE-Pyr (Fig 5), suggesting that the peptides form pores according to the toroidal pore model. This result is in accordance with a study making use of lipid vesicles containing the toxins and the fluorescent probe PC-6-NBD. Quenching of the probe’s fluorescence by albumin in the bulk aqueous phase demonstrated toxin-triggered lipid redistribution between bilayer leaflets, suggesting formation of a toroidal pore [50]. Furthermore, ^31^P NMR studies of the interaction between Eqt II and multilamellar lipid vesicles indicated formation of non-lamellar lipid, also in agreement with the toroidal pore model [19]. In studies with StII_1-30_ [24] and with the full-length toxins [59], it was found that the hydrodynamic pore radius is approx. 1 nm in both cases.

The topography of toroidal pores is described as consisting of peptides, or protein domains, organized as amphipathic α-helices lying approximately parallel to the membrane surface. In order to form the pore, several peptide molecules would recruit lipids that favor membrane positive curvature. We also suggest that the amino acid sequences in the peptides encode the ability to recruit these lipids, thus different sequences would possess different ability to promote pore formation. In the pore, the helices polar faces are interspersed with phospholipid head groups, lining up the pore lumen; the whole ensemble assumes positive curvature. Such molecular arrangement has been proposed for the N-terminal amphipathic α-helices of St I, St II, and Eqt II [44]. Indeed, Ros et al. [27, 28], making use of the procedure of Eisenberg et al. [60] to establish the location of the amphipathic α-helices of these proteins, as well as that of fragaceatoxin C, found that these sequences do have a propensity to lie at the membrane surface.

### Peptide location in micelles and bilayers represents peptide topography in different pore forming steps

Micelles and LUV or larger vesicles have different geometries and molecular organization of their lipid constituents, thus leading to different interactions with, and therefore, different topographical arrangements of, amphipathic peptides. Differences in packing, interface degree of hydration, and exposure of the hydrocarbon chains, are probably, the contributing factors for the differences in peptide-membrane affinity and in peptide-lipid topographical organization.

The positive curvature in micelles leads to looser molecular packing and more exposed and more hydrated acyl chains. These features favor the contribution of hydrophobic interactions between the micellar hydrophobic environment and the peptide helix nonpolar face, thus enhancing its membrane affinity, and decreasing the requirement of electrostatic interactions for binding (Table 4). Binding to bilayers requires electrostatic interactions between negatively charged lipid head groups and positively charged peptide residues. The greater molecular packing in bilayers causes the lipid acyl chains to be less exposed for direct interaction with the peptide’s hydrophobic face. Before accommodation of the amphipathic α-helix in the bilayer hydrophilic/hydrophobic interface, contribution of electrostatic forces is necessary for the initial stabilization of peptide-lipid interaction.

Binding of sticholysins N-terminal peptides to bilayers can be envisioned as the initial step in the mechanism of pore formation, comprising acquisition of secondary structure and helix interaction with the water-membrane interface. As proposed by Bozelli et al. [61] and Santos et al. [62], due to the looser packing of lipids in micelles, which possibly resembles that in the toroidal pore structure, where the lipids are in positive curvature, the interaction with micelles would mimic the topography of the toxin’s N-terminal segment in the toroidal pore region. The differences between bilayer-bound and micelle-bound peptides helical content (Table 2) would, then, be related to the peptides conformation at the membrane surface and in the pore region, respectively. Also, the greater affinity for micelles (Table 3) would be related to the pore formation mechanism and the maintenance of the N-terminal helices of Sts in the pore structure.

### Peptides activity: LUV permeabilization and hemolysis

In previous studies Ros et al [26, 28] examined the permeabilizing activity of the longer peptides StI_1-31_ and StII_1-30_ towards LUV of PC:SM 50:50, and compared the results to those obtained for the whole toxins. The hemolytic activity of the peptides and proteins was also assessed. In addition, the activity of StII_1-30_ upon LUV consisting of PC:SM:PA 50:45:5 was also studied [26]. Data for the toxins are presented in Table 4, in conjunction with the results obtained in the present work aiming at dissecting the role of the N-terminal α-helix on the toxins mechanism of action.

The leakage studies clearly demonstrated that higher concentrations of StI_12-31_ and StII_11-30_ than of their longer counterparts are required to promote membrane permeabilization, and that these concentrations are orders of magnitude higher than those of the whole toxins. This behavior is expected since other protein regions play a role in membrane binding and in favoring pore formation, especially the first ten residues in the N-terminus. In addition, a cluster of aromatic amino acid residues anchors the body of the protein to the membrane interface, providing a binding site for the phosphocholine moiety of phosphatidylcholine, and, very likely, of sphingomyelin, known to be important for actinoporin binding. These interactions contribute to high membrane-toxin affinity [4, 44].

The LUV permeabilization (Fig 6, Table 4) and hemolysis (Fig 7) studies with the N-terminal peptides evinced the importance of the 1–11 (1–10) residues for activity. With regard to hemolysis, while the StII_1-30_ peptide was active against RBC (Fig 7), StI_1-31_ had no effect [28]. In addition, the short peptides were essentially not active in the same concentration range (Fig 7). These results are probably due to the fact that the 1–10 segment in St II contains a high proportion of hydrophobic amino acids that assist the N-terminal helix in anchoring the toxin to the membrane and may have a role in reorganizing the lipids involved in pore formation. Indeed, this sequence was found to have a propensity to have a transmembrane orientation. In contrast, the 1–11 stretch in St I is much more polar, showing a propensity to remain at the membrane surface [26]. This notion is corroborated by CF leakage experiments (Table 4) that show that (P/L)_50_ ratios differ slightly between StI_1-31_ and StI_12-31_, whereas this ratio is ca. six times smaller for StII_1-30_ than for StII_11-30_. In addition, when comparing both long peptides, it is seen that the (P/L)_50_ ratio is twelve times higher for StI_1-31_ than for StII_1-30_. Thus, differences in the extent of the effect observed for the toxins, as well as for full length N-terminal peptides would be at least partly due to the different hydrophobicity of the first eleven (ten) residues.

As for the short peptides, that contain the amphipathic α-helices of St I and St II, but do not contain the first eleven (ten) N-terminal residues, it is worth noticing that binding to micelles and bilayers fits an approximately 1:1 stoichiometry (Fig 3), suggesting that the peptides bind essentially in the monomeric form. In contrast, analysis of the Hill coefficient (n, Table 4) obtained from fittings of leakage experiments (Fig 6) indicated that this process involves positive cooperativity, in agreement with the hypothesis of formation of an oligomeric pore. Table 4 shows that, while the values of n for the St II peptides, vary between 3.0 and 4.4, those for StI_12-31_ vary from 1.7 to 2.5. Since the early work of Belmonte et al. [63] various studies have suggested that there is no fixed stoichiometry in the pore; rather there is a distribution in the number of monomers giving rise to this structure [21, 22, 64]. It is conceivable that the difference between the values of Hill coefficients for StI_12-31_ and StII_11-30_ (and its TOAC analogue) would be related to different pore stoichiometry. In this context, the smaller number of monomers forming the StI_12-31_ pore could also be partly responsible for the peptide’s lesser activity.

The fact that the short peptides are still capable of promoting LUV permeabilization provides evidence for the ability of this region to form the lining part of the pore. However, the higher (P/L)_50_ ratios, when compared to the respective long peptides reinforces the notion that the first residues play the role of anchoring the pore in the membrane (Table 4). Interestingly, albeit the difference between (P/L)_50_ ratios for the shorter peptides is smaller than that between the longer peptides, a difference between their permeabilizing activity is still observed, demonstrating that the higher effectiveness of St II is not only due to the higher hydrophobicity of its 1–10 sequence, but is also partly due to the helix sequence itself, since StII_11-30_, as well as its TOAC derivative, are more effective than StI_12-31_. Thus, in the search to understand the events and structural protein features involved in the mechanism of pore formation by actinoporins, the present work presents evidence pointing to the role of the N-terminal amphipathic α-helix in this process.

## Conclusions

Several approaches were employed to study peptides corresponding to the N-terminal amphipathic α-helices of the sea anemone actinoporins sticholysin I and sticholysin II. The work was based on the hypothesis that peptide fragments can reproduce the structure and function of these segments in the entire toxins. Indeed, molecular modeling and CD spectra of micelle- and bilayer vesicle-bound peptides showed that these regions form amphipathic α-helices, as found in high resolution structures of the whole proteins. Fluorescence quenching studies evinced that the N-terminus of a paramagnetically-labeled peptide analogue was located at the membrane interface. Phospholipid inter-leaflet redistribution suggested toroidal pore formation, in agreement with the mechanism of action proposed for the toxins. Differences between bilayers and micelles in head group packing and in curvature promoted different peptide-membrane interactions, leading to the proposal that the peptides topography in micelles resembles that of the toxins in the toroidal pore. And finally, the peptides mimicked the toxins permeabilizing activity, St II peptides being more effective than StI_12-31_.

This ensemble of results supports the notion that the formation of a toroidal pore implies specificity from the point of view of membrane lipid composition, *i*.*e*, the membrane should be able to provide lipids with propensity to form positive curvature. On the other hand, the pore lining α-helical sequence must encode the ability to recruit these lipids and to adjust to the pore positive curvature. In the present study, since the membrane-forming lipids were the same, we conclude that the pore-forming ability does rely on the peptides sequence, StII_11-30_ being more effective in binding and inducing solute leakage, in analogy to the behavior of the respective toxins. It has already been shown that the first 10 residues of actinoporins are important for their mechanism of action. To our knowledge, this is the first time that the N-terminal α-helix amino acid sequence is demonstrated to play a role in the binding and lytic activity of these toxins.

## Supporting information

S1 Fig Atomic representation of the models of peptides. StI_12-31_ (A) and StII_11-30_ (B) obtained by the PEP-FOLD program highlighting the positive (blue) and negative (red) side chains able to establish ionic pairs. Residues types: Non-polar (black), polar uncharged (green).
(TIF)

S2 Fig CD spectra of the peptides bound to micelles. StI_12-31_ (A), StII_11-30_ (B) and N-TOAC-StII_11-30_ (C) in solution and in presence of 10 mM of lysophospholipids of variable composition.
(TIF)

S3 Fig CD spectra of N-TOAC-StII_11-30_ bound to gel phase membranes. CD spectra of N-TOAC-StII_11-30_ in solution and in presence of variable concentrations of LUV of DPPC (A) and DPPC:DMPA (90:10) (B).
(TIF)
