# Supplementary figures and images for: Dissecting the mechanism of action of actinoporins. Role of the N-terminal amphipathic α-helix in membrane binding and pore activity of sticholysins I and II
Source: PLoS One. 2018 Aug 30;13(8):e0202981. doi: 10.1371/journal.pone.0202981 (PMC6117003; doi:10.1371/journal.pone.0202981)

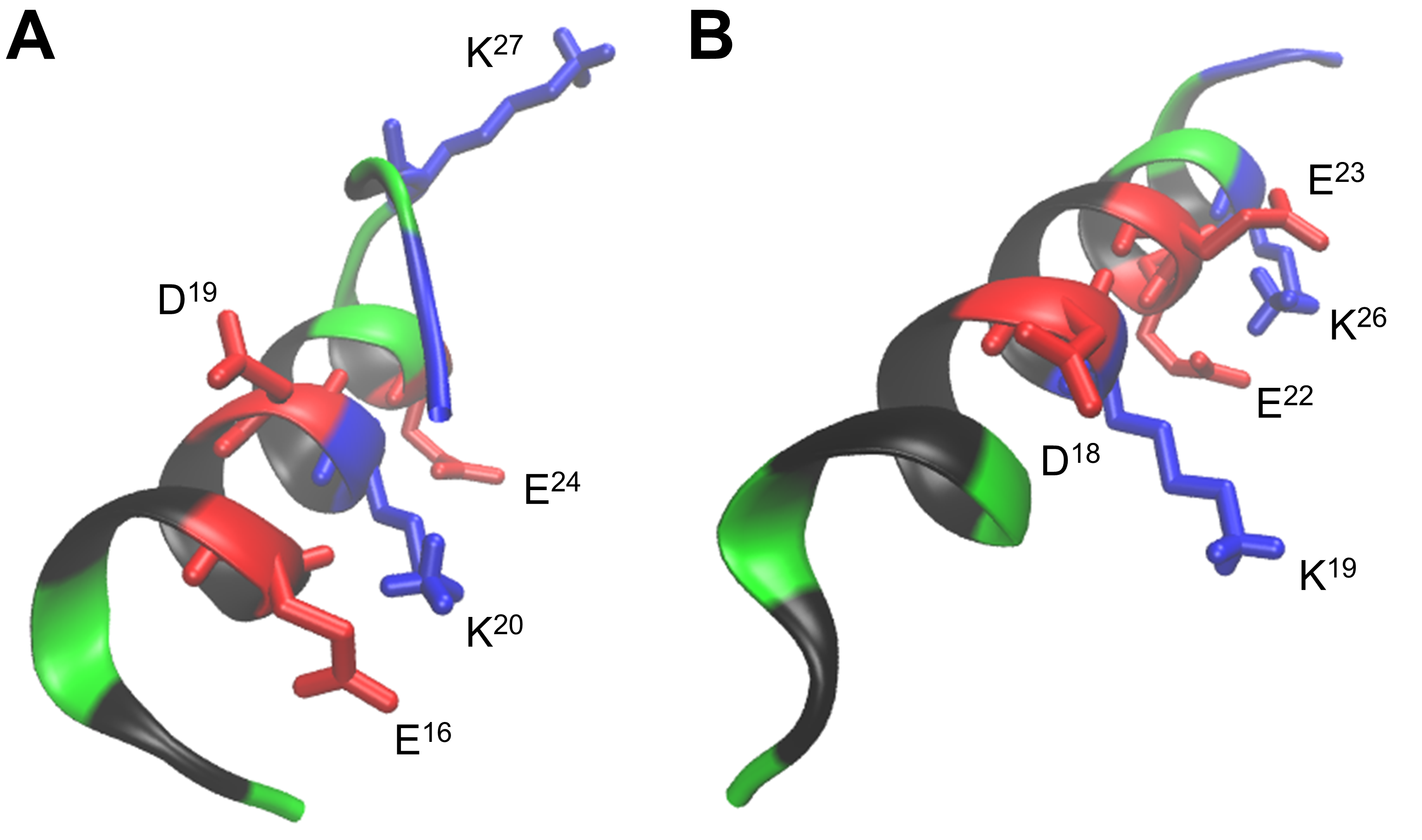

Supplement: S1 Fig — StI12-31 (A) and StII11-30 (B) obtained by the PEP-FOLD program highlighting the positive (blue) and negative (red) side chains able to establish ionic pairs. Residues types: Non-polar (black), polar uncharged (green). (TIF) [file pone.0202981.s001.tif]

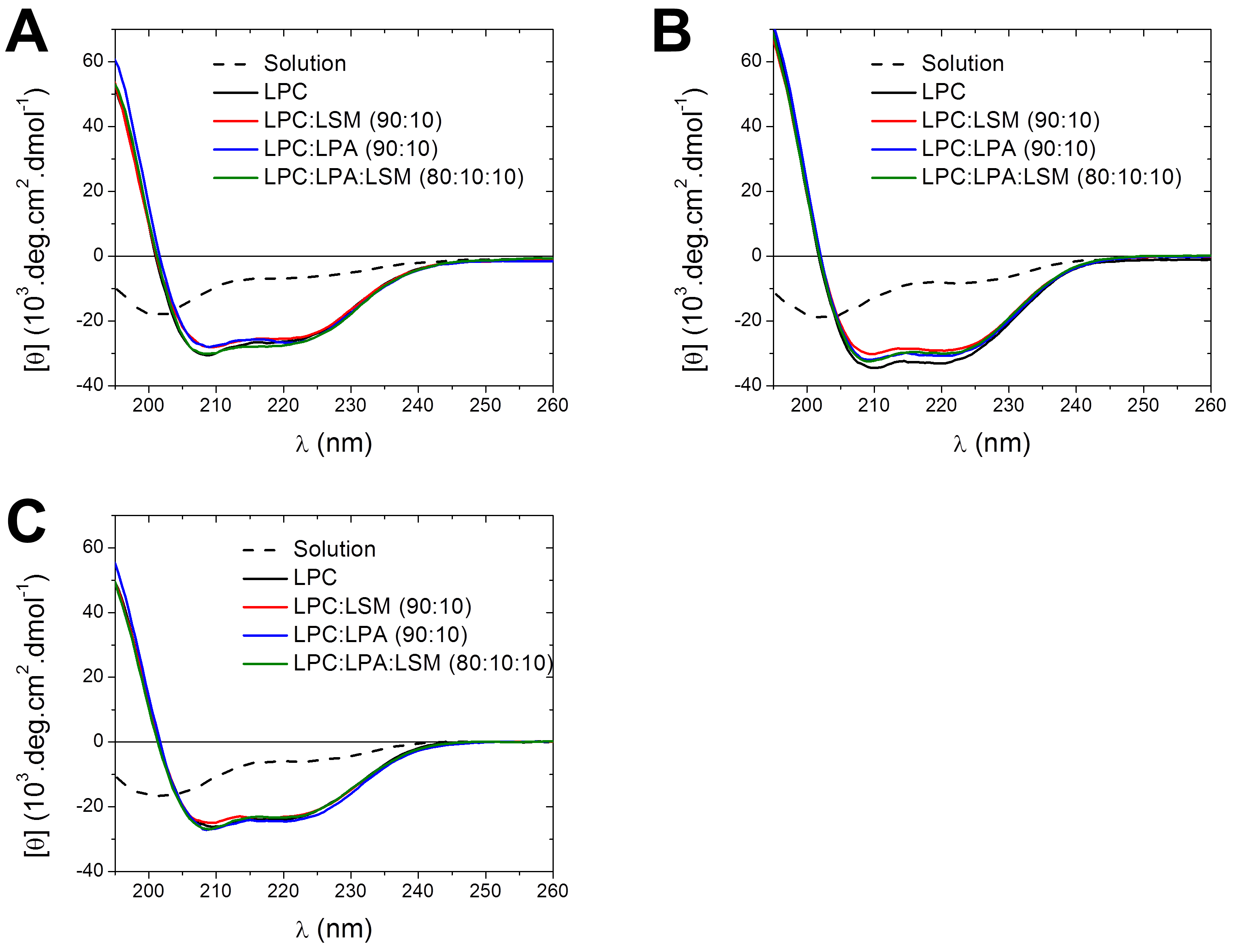

Supplement: S2 Fig — StI12-31 (A), StII11-30 (B) and N-TOAC-StII11-30 (C) in solution and in presence of 10 mM of lysophospholipids of variable composition. (TIF) [file pone.0202981.s002.tif]

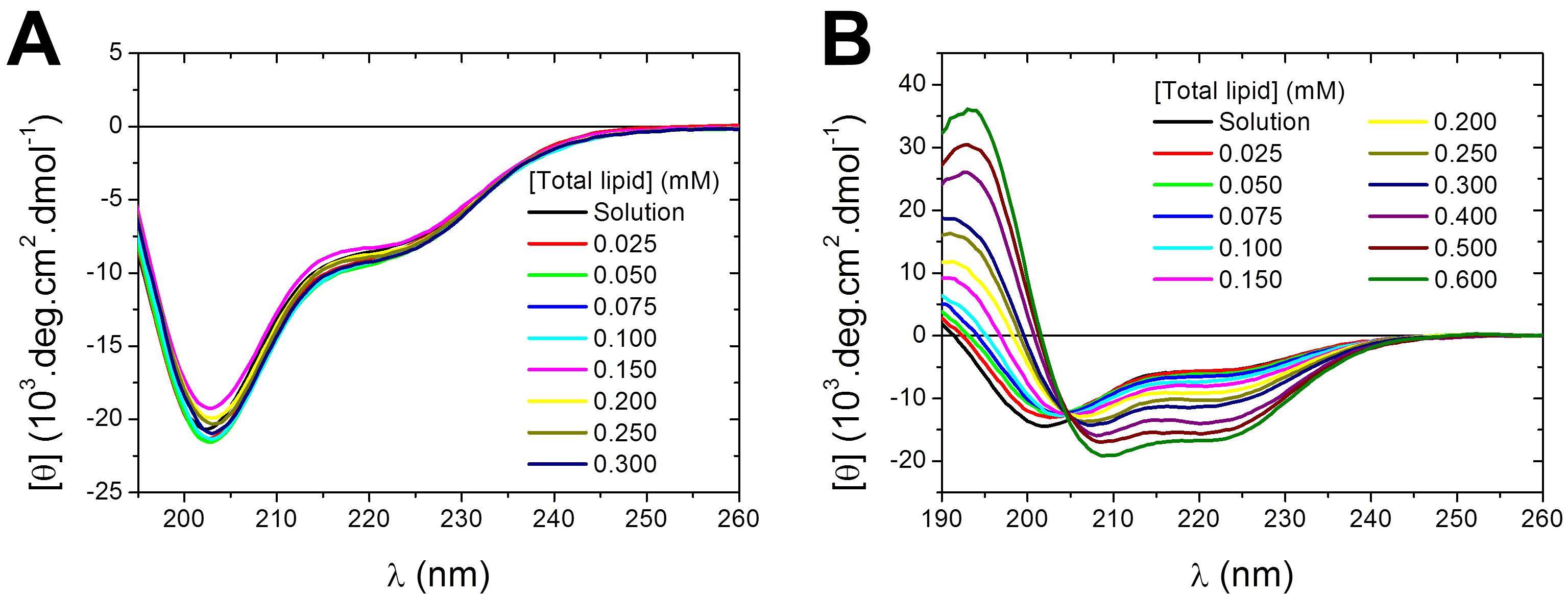

Supplement: S3 Fig — CD spectra of N-TOAC-StII11-30 in solution and in presence of variable concentrations of LUV of DPPC (A) and DPPC:DMPA (90:10) (B). (TIF) [file pone.0202981.s003.tif]
